# Improvement of XYL10C_∆N catalytic performance through loop engineering for lignocellulosic biomass utilization in feed and fuel industries

**DOI:** 10.1186/s13068-021-02044-3

**Published:** 2021-10-01

**Authors:** Shuai You, Ziqian Zha, Jing Li, Wenxin Zhang, Zhiyuan Bai, Yanghao Hu, Xue Wang, Yiwen Chen, Zhongli Chen, Jun Wang, Huiying Luo

**Affiliations:** 1 School of Biotechnology, Jiangsu University of Science and Technology, Zhenjiang, 212018 People’s Republic of China; 2 Sericultural Research Institute, Chinese Academy of Agricultural Sciences, Zhenjiang, 212018 People’s Republic of China; 3 Department of Nephrology, Affiliated Hospital of Jiangsu University, Zhenjiang, 212001 People’s Republic of China; 4 Xinyuan Cocoon Silk Group Co., Ltd., Nantong, 226600 People’s Republic of China; 5 Institute of Animal Sciences, Chinese Academy of Agricultural Sciences, Beijing, 100081 People’s Republic of China

**Keywords:** GH10 xylanase, Low-temperature catalytic performance, Protein engineering, Synergism, Biomass degradation

## Abstract

**Background:**

Xylanase, an important accessory enzyme that acts in synergy with cellulase, is widely used to degrade lignocellulosic biomass. Thermostable enzymes with good catalytic activity at lower temperatures have great potential for future applications in the feed and fuel industries, which have distinct demands; however, the potential of the enzymes is yet to be researched.

**Results:**

In this study, a structure-based semi-rational design strategy was applied to enhance the low-temperature catalytic performance of *Bispora* sp. MEY-1 XYL10C_∆N wild-type (WT). Screening and comparisons were performed for the WT and mutant strains. Compared to the WT, the mutant M53S/F54L/N207G exhibited higher specific activity (2.9-fold; 2090 vs. 710 U/mg) and catalytic efficiency (2.8-fold; 1530 vs*.* 550 mL/s mg) at 40 °C, and also showed higher thermostability (the melting temperature and temperature of 50% activity loss after 30 min treatment increased by 7.7 °C and 3.5 °C, respectively). Compared with the cellulase-only treatment, combined treatment with M53S/F54L/N207G and cellulase increased the reducing sugar contents from corn stalk, wheat bran, and corn cob by 1.6-, 1.2-, and 1.4-folds, with 1.9, 1.2, and 1.6 as the highest degrees of synergy, respectively.

**Conclusions:**

This study provides useful insights into the underlying mechanism and methods of xylanase modification for industrial utilization. We identified loop2 as a key functional area affecting the low-temperature catalytic efficiency of GH10 xylanase. The thermostable mutant M53S/F54L/N207G was selected for the highest low-temperature catalytic efficiency and reducing sugar yield in synergy with cellulase in the degradation of different types of lignocellulosic biomass.

**Graphic Abstract:**

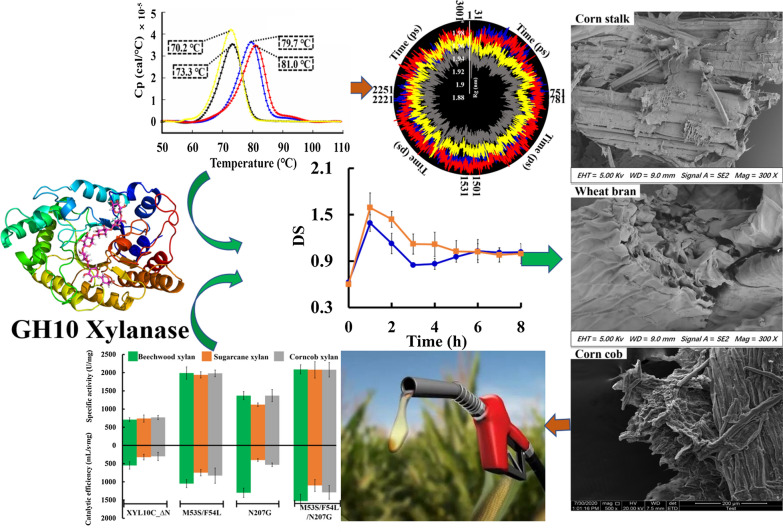

**Supplementary Information:**

The online version contains supplementary material available at 10.1186/s13068-021-02044-3.

## Highlights

1. Key sites were proved to promote low-temperature catalytic efficiency of xylanase.
2. Thermostable mutant with improved catalytic efficiency at 40 °C was obtained.
3. Synergistic hydrolysis was dramatically improved between the mutant and cellulase.
4. Agricultural wastes were converted into fermentable sugars by enzymolysis.

**Supplementary Information:**

The online version contains supplementary material available at 10.1186/s13068-021-02044-3.

## Background

Lignocellulosic biomass is considered an asset in the production of renewable energy because it is present in abundance, inexpensive, and eco-friendly [1, 2]. The conversion of renewable lignocellulosic materials into high value-added products through enzymatic decomposition is a sustainable and promising technology for industrial production [3, 4]. This process requires the cooperative action of cellulolytic and xylanolytic enzymes, of which endo-cellulase and endo-xylanase are the main enzymes that hydrolyze the primary chains of cellulose and xylan, respectively [5]. Research efforts on the bioconversion of lignocellulosic biomass into biofuels have focused on the development of enzymes with unique properties and suitability for cost-effective optimization processes [6].

Xylanase, as an important accessory enzyme, is widely used in biomass degradation and in the feed and fuel industries [7]. For the feed industry, the ideal enzyme is expected to possess the following features: (1) excellent thermal stability to resist the high-temperature pelleting process, and (2) high catalytic activity at animal body temperatures (~ 40 °C) [8]. Meanwhile, enzymes with similar characteristics could be competitive for the biofuel industry, based on their stability at high temperatures for the maximum decomposition of biomass as well as their ability to maintain reactivity at lower temperatures to realize the ultimate goal of low energy consumption [9]. Many studies have been conducted on the underlying mechanism and improvement of enzyme thermostability [10]. However, there are relatively few reports on the augmentation of the catalytic efficiency of xylanase [11–15]. Almost no studies have reported the improvement of catalytic activity of thermotolerant xylanases at lower temperatures. Thus, as a potential area of research for future applications, the catalytic activity of thermostable enzymes at lower temperatures could be investigated.

XYL10C_∆N, an N-terminal-truncated mutant of XYL10C from *Bispora* sp. MEY-1 [16], was used as the study material. The material, with a crystal structure, is characterized by high heat resistance and catalytic efficiency under high-temperature conditions. However, its specific activity at 40 °C was less than one-tenth of its maximum catalytic activity [17]. In this study, XYL10C_∆N was modified to improve its catalytic activity at 40 °C while retaining its thermostability.

## Results and discussion

### Selection of the mutation site and site-directed mutagenesis

**Fig. 1:**
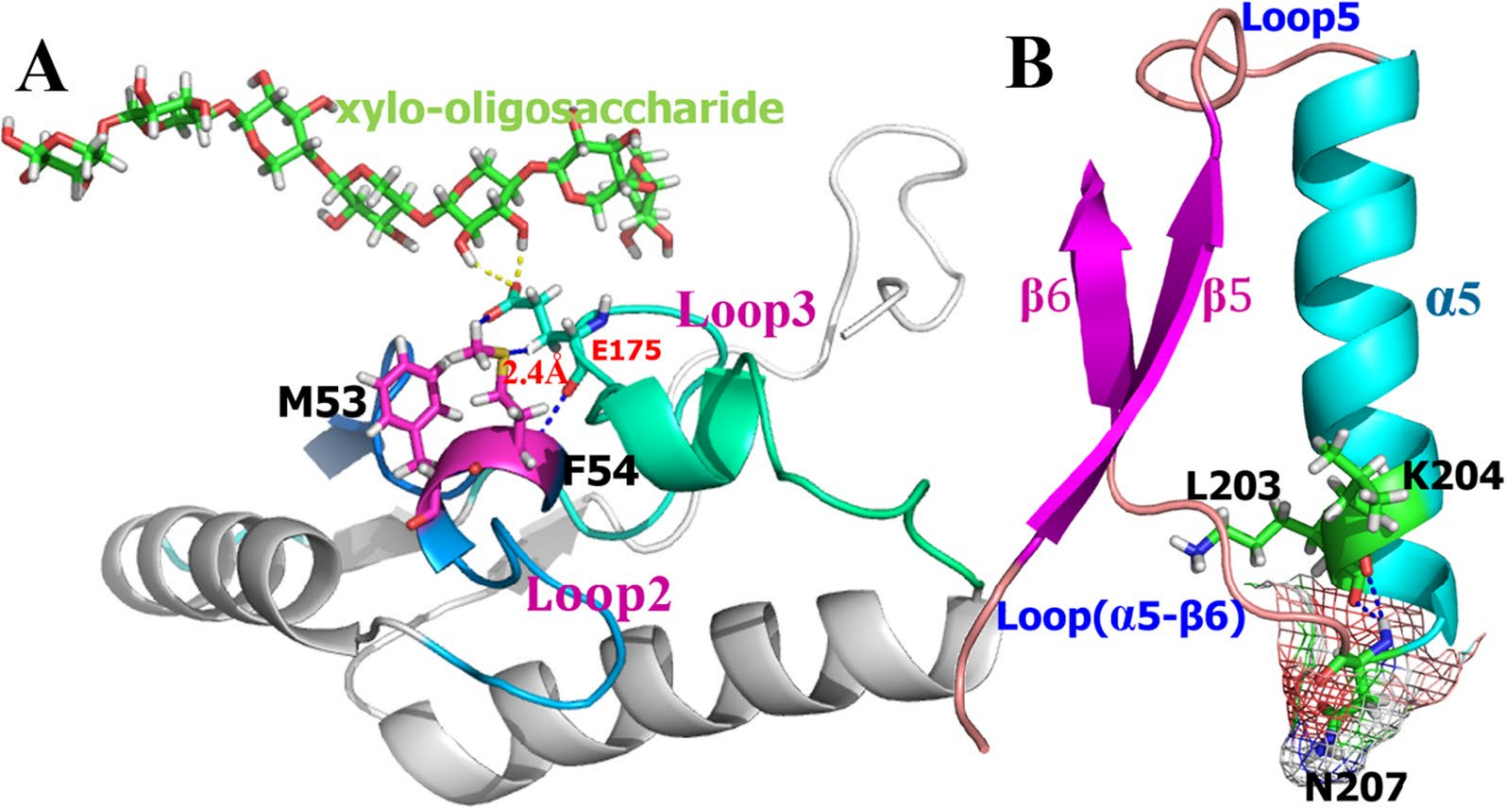
Selection of sites of mutation. **A** Location and configuration of Met53 and Phe54; **B** location and configuration of Asn207

The loop regions of the TIM-barrel enzyme are considered invaluable for enzyme–substrate interactions [18]. Met53 and Phe54, located in loop2, form the only helical segment in this random coil. We have previously demonstrated that Glu91 in loop3 affects the binding of xylanases of the GH10 family to their substrates [17]. In other words, when Glu is present at the site, the enzyme shows the strongest binding force with the substrate, highest catalytic efficiency, and best thermostability. As shown in Fig. 1A, the shortest distance between Phe54 and Glu91 was 2.4 Å. Based on this, we speculated that mutations at the two sites might affect the conformational changes in Glu91, and thus, enzyme catalysis. Sequence alignment analysis showed that even though these two amino acids were not highly conserved, there were only a few noticeable changes (Additional file 2: Figure S1). As a result, Met53/Phe54 was simultaneously mutated into alternate groups of amino acids by site-directed mutagenesis, as shown in Additional file 1: Table S1. The catalytic activities of the different mutants were assessed at 40 °C. Sequence evolution and three-dimensional conformation analysis indicated that the Asn207 residue of XYL10C_∆N wild-type (WT) (PDB entry: 5XZO/5XZU) [12] may be the key switch residue for controlling the movement of loop (α5-β6) and loop5 (Fig. 1B). The catalytic performance of the WT may be related to the conformational plasticity of the flexible structure. Therefore, based on the data on amino acid evolution, Asn207 was confirmed to be mutated to Gly207.

### Production and purification of recombinant XYL10C_∆N and its mutants

The WT enzyme and its mutants were successfully expressed in *P. pastoris* GS115 and purified. Except for the mutants MF53/54AA and MF53/54VV, which displayed no activity, enzyme activity was detected in all other mutants. After ion-exchange purification, as described in the Materials and methods, M53S/F54L and N207G showed significant improvement in specific activities compared to the WT at 40 °C (1590 and 1000 U/mg vs. 710 U/mg, respectively) (Additional file 2: Figure S2). Thus, the single (M53S/F54L and N207G) and combined (M53S/F54L/N207G) mutants were expressed and characterized as described above.

Sodium dodecyl sulfate-polyacrylamide gel electrophoresis (SDS-PAGE) analysis showed that the electrophoretic purity of all enzymes was greater than 95%, and the molecular masses ranged from 45 to 65 kDa, which were greater than the theoretical value (approximately 38.2 kDa) (Additional file 2: Figure S3). A single band was observed for all enzymes after treatment with Endo H, which corresponded to the theoretical molecular weight.

### Comparison of the pH properties and temperature optima of the WT and mutants

**Fig. 2:**
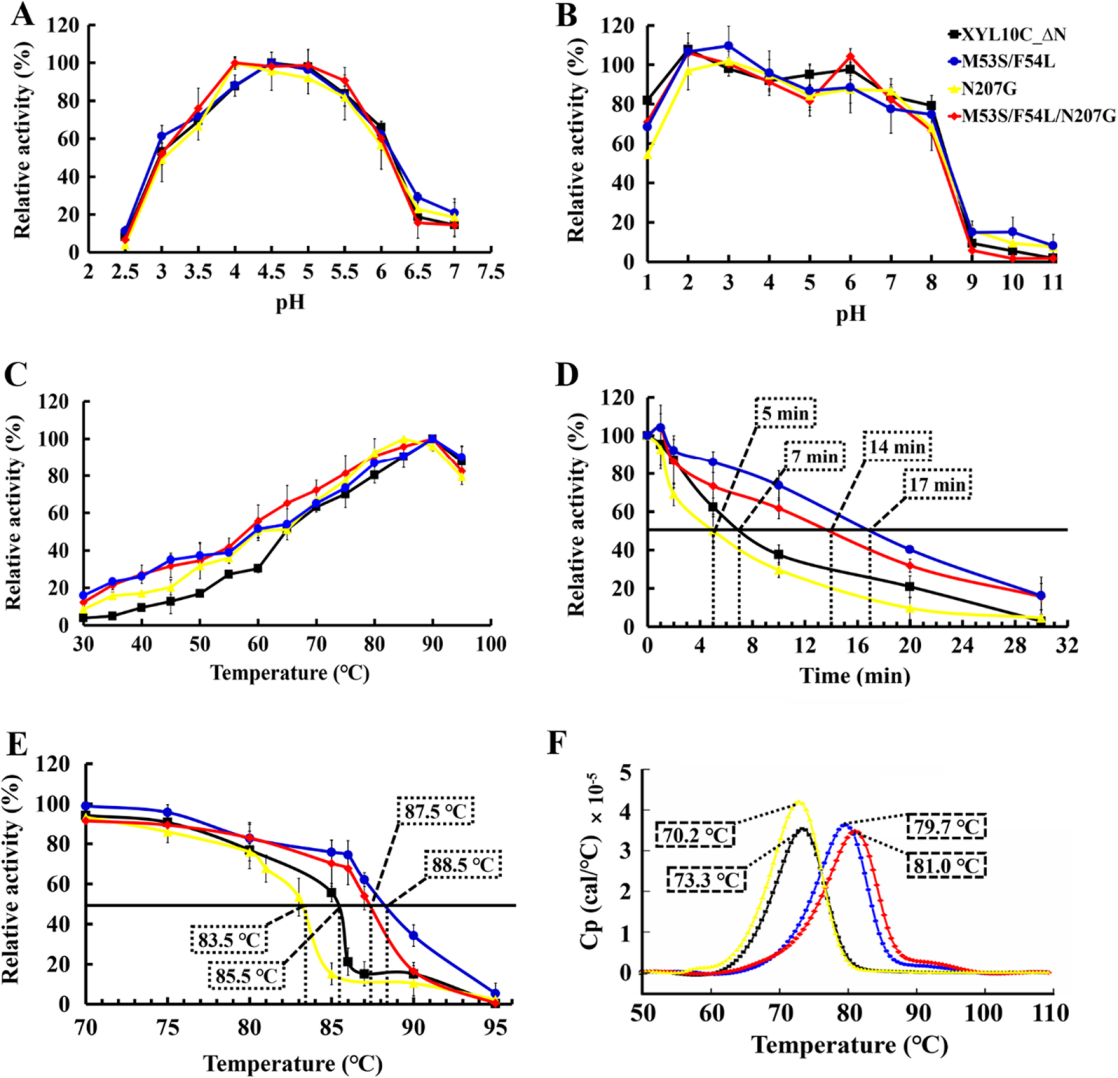
Properties of the purified recombinant wild-type XYL10C_∆N and its mutants. **A** pH–activity profiles of each enzyme tested at the respective optimal temperatures; **B** pH–stability profiles. After the enzymes were incubated for 1 h at 37 °C in buffers with pH ranging from 1.0 to 11.0, the residual activities were determined in 100 mM McIlvaine buffer at the optimal temperature and pH of each enzyme; **C** temperature–activity profiles tested at the optimal pH of each enzyme; **D** half-lives of wild-type XYL10C_∆N and its mutants at 90 °C; **E** Temperature stability profiles (*T*_50_). Enzyme inactivation assay at 70–95 °C for 30 min; **F** thermograms obtained using differential scanning calorimetry. The calorimetric recordings of 200 µg/mL XYL10C_∆N and mutants were scanned at 2 °C/min in 20 mM McIlvaine buffer (pH 6.5)

Using beechwood xylan as the substrate, the effect of pH on the activity and stability of all enzymes was determined at 85 °C. As shown in Fig. 2A, the optimum pH for WT and its mutants was 4.0 or 4.5. This result was similar to that of GH10 xylanase obtained from most fungi, such as *Penicillium canescens* [19], *Trichoderma reesei* [20], and *Aspergillus nidulans* [21]. The relative activities of the enzymes did not differ significantly in the same pH environment. All enzymes maintained more than 50% relative activity between pH 3.0 and 6.0. Notably, the pH range of the combination mutant M53S/F54L/N207G was wider, which facilitated the maintenance of more than 76% relative enzyme activity between pH 3.5 and 5.5. This was higher than the relatively activity achieved for the WT and the two mutants. In terms of pH stability, all mutants were similar to the WT, and all enzymes maintained > 50% of their maximal activity at pH values ranging from 1 to 8 after incubation at 37 °C for 1 h (Fig. 2B). The excellent stability of all mutants under acidic conditions highlights their suitability for application in bioethanol, detergent, and feed additive industries [7].

The temperature corresponding to the maximum activity (*T*_max_) of the enzymes was determined over a wide range of temperatures (from 40 to 95 °C) at the respective optimal pH values, using beechwood xylan as the substrate. Notably, similar to that of 1VBU from *Thermotoga maritima* [22], the *T*_max_ of the WT, M53S/F54L, and M53S/F54L/N207G was 90 °C (Fig. 2C).

Notably, the steady improvement of enzyme activity was observed in the mutants at lower temperatures than in the WT. For example, at 60 °C, all mutants showed 51–55% of their maximal activity, which was considerably higher than the result obtained for the WT (~ 30% of its maximal activity). When the temperature decreased to 40 °C, M53S/F54L, N207G, and M53S/F54L/N207G maintained 27%, 17%, and 26% of their activities, respectively, whereas the WT maintained only 9% activity. The relative activities of the three mutants were 1.9–3.0 times higher than that of the WT. This indicates that mutations at these sites increase the catalytic activity of enzymes at low temperatures. As the temperature of the digestive tract of animals is maintained at approximately 40 °C, the increase in the relative activities of the mutant enzymes at 40 °C is more conducive to the application of xylanase in feed supplement production.

### Kinetics and specific activity of the WT and its mutants at 40 °C

Research on GH10 xylanases has primarily focused on heat resistance mechanisms and molecular improvement, and studies on the catalytic mechanism are relatively rare [23]. However, Xiong et al. mutated the N86 residue of the − 2 substrate-binding region of GH10 xylanase to Gln, which enhanced the hydrogen bond interaction with the surrounding amino acids, increased the volume of the catalytic pocket, and eventually increased the catalytic activity of the enzyme by a factor of 1.25 [24]. Through virtual mutation and molecular dynamics (MD) simulation, Wu et al. introduced Glu and Asn into the catalytic center of xylanase, which enhanced the binding force between the substrate molecule and the catalytic channel, and eventually increased the catalytic efficiency of the enzyme by 72% [25]. In addition, previous studies have primarily focused on changes in the catalytic efficiency of enzymes at the optimal temperature [26]. However, no study has been conducted on the improvement of catalytic activity at a specific application temperature [10].

The kinetic parameters of the enzyme were determined using beechwood xylan as the substrate at 40 °C. The graphs for the equations derived from the Lineweaver–Burk regression plots, which were used to calculate the kinetic parameters of each enzyme, are included in the Additional file 2: Figure S4. As shown in Table 1, compared with that of the WT, the *K*_m_ values of the mutants N207G and M53S/F54L/N207G were lower (0.85 and 0.83 vs. 0.96 mg/mL, respectively), indicating that the binding force of the two mutants to the substrate was slightly greater than that of the WT. However, the *K*_m_ value of the mutant M53S/F54L increased compared to that of the WT (1.30 vs. 0.96 mg/mL). Notably, the product release rate (*k*_cat_) of the three mutants was higher than that of the WT, suggesting that all mutants were superior to the WT in terms of the substrate turnover number. Consequently, the catalytic efficiencies (*k*_cat_/*K*_m_) of the two mutants and their combination improved considerably (by 1.9–2.8 fold) compared to that of the WT, and the catalytic efficiency of the combined mutant M53S/F54L/N207G exceeded 1500 mL/s·mg, which was primarily attributed to the enhanced binding force between the enzyme and the substrate and the rapid release of the product. In addition, compared with the WT, all mutants showed improvement of specific activities (by 1.9–2.9-fold), and the specific activity of the mutant M53S/F54L/N207G exceeded 2000 U/mg, which was higher than those of xylanases from *Bacillus* sp. [23], *Aspergillus aculeatus*, and *Aspergillus niger* [27] at the same temperature.

**Table 1:** Kinetic parameters and specific activity of xylanases against beechwood xylan at 40 °C

	*K*_m_ (mg/mL)	*k*_cat_ (s^−1^)	*V*_max_ (μmol/min·mg)	*k*_cat_/*K*_m_ (mL/s·mg)	Specific activity (U/mg)
XYL10C_∆N	0.96 ± 0.18	530 ± 40	830 ± 62	550 ± 60	710 ± 45
M53S/F54L	1.30 ± 0.10	1090 ± 44	2140 ± 69	1050 ± 31	1990 ± 77
N207G	0.85 ± 0.01	1110 ± 6	1750 ± 9	1300 ± 11	1370 ± 64
M53S/F54L/N207G	0.83 ± 0.10	1260 ± 44	1980 ± 70	1530 ± 144	2090 ± 197

The kinetic values are shown as means ± standard deviations (*n* = 3)

The same trends in specific activity and catalytic efficiency were observed when sugarcane xylan and corn cob xylan were used as substrates. For example, as shown in Table 2, the three mutants showed 1.5–2.8 times and 1.3–3.4 times higher specific activities and catalytic efficiencies, respectively, than the WT, when sugarcane xylan was used as the substrate. The mutants showed 3–5 times and 0.8–1.7 times higher specific activities and catalytic efficiencies, respectively, than the WT, when corncob xylan was used as the substrate. It is worth noting that the catalytic efficiencies and specific activities of the combined mutants were the highest. When sugarcane xylan and corncob xylan were used as substrates, the specific activity was 2080 U/mg for both, and the catalytic efficiencies were 1100 and 1290 μmol/min·mg, respectively.

**Table 2:** Kinetic parameters and specific activity of xylanases against sugarcane and corncob xylan at 40 °C

Substrate	Sugarcane xylan				Corncob xylan			
Enzymes	*K*_m_ (mg/mL)	*V*_max_ (μmol/min·mg)	*k*_cat_/*K*_m_ (mL/s·mg)	Specific activity (U/mg)	*K*_m_ (mg/mL)	*V*_max_ (μmol/min·mg)	*k*_cat_/*K*_m_ (mL/s·mg)	Specific activity (U/mg)
XYL10C_∆N	1.52 ± 0.04	730 ± 38	320 ± 28	740 ± 33	1.89 ± 0.14	810 ± 52	300 ± 15	770 ± 63
M53S/F54L	1.63 ± 0.17	1920 ± 81	750 ± 41	1940 ± 77	1.44 ± 0.16	1880 ± 88	830 ± 48	1980 ± 91
N207G	1.91 ± 0.08	1210 ± 56	400 ± 33	1120 ± 82	1.55 ± 0.11	1290 ± 72	530 ± 47	1370 ± 62
M53S/F54L/N207G	1.18 ± 0.10	2030 ± 79	1100 ± 81	2080 ± 85	1.03 ± 0.04	2090 ± 84	1290 ± 81	2080 ± 92

The kinetic values are shown as means ± standard deviations (*n* = 3)

### Thermostability assays of the WT and mutants

In general, the rigidity of the protein structure corresponds to the stability of the enzyme, and greater protein structure flexibility usually corresponds to greater catalytic effectiveness. [28]. Thus, it is challenging to design enzyme molecules to enhance the catalytic performance of enzymes without reduction of the original level of thermostability. For example, Wang et al. improved the melting temperature (*T*_m_) of *Streptomyces* sp. strain S9 xylanase by 7.0 °C, but the catalytic activity was lowered to 75% [29]. We evaluated the thermostability of all dominant mutants. As shown in Fig. 2D, the half-lives (*t*_1/2_) of the WT, M53S/F54L, N207G, and M53S/F54L/N207G, at 90 °C, were 7, 17, 5, and 14 min, respectively. The results showed that the mutants could be arranged as M53S/F54L > M53S/F54L/N207G > XYL10C_∆N > N207G in decreasing order of thermostability. In addition, the temperature at which 50% activity is lost after 30 min treatment (*T*_50_) and the *T*_m_ of the enzymes also indicated that the mutants M53S/F54L and M53S/F54L/N207G were more heat-resistant than the WT. The results showed that the *T*_50_ values of the mutants M53S/F54L and M53S/F54L/N207G were 3 °C and 2 °C higher (Fig. 2E), and the *T*_m_ values were 7.7 °C and 6.4 °C higher, compared to those of the WT, respectively (Fig. 2F). The excellent thermal stability under high temperature makes xylanase more suitable for applications in the feed, food, biofuel, and biotransformation industries. These results also indicate that the combinatorial mutant M53S/F54L/N207G showed higher activity at lower temperatures and maintained greater residual activity after high-temperature treatment.

### Diverse mechanisms underlying the improvement of the characteristics of the mutants

**Fig. 3:**
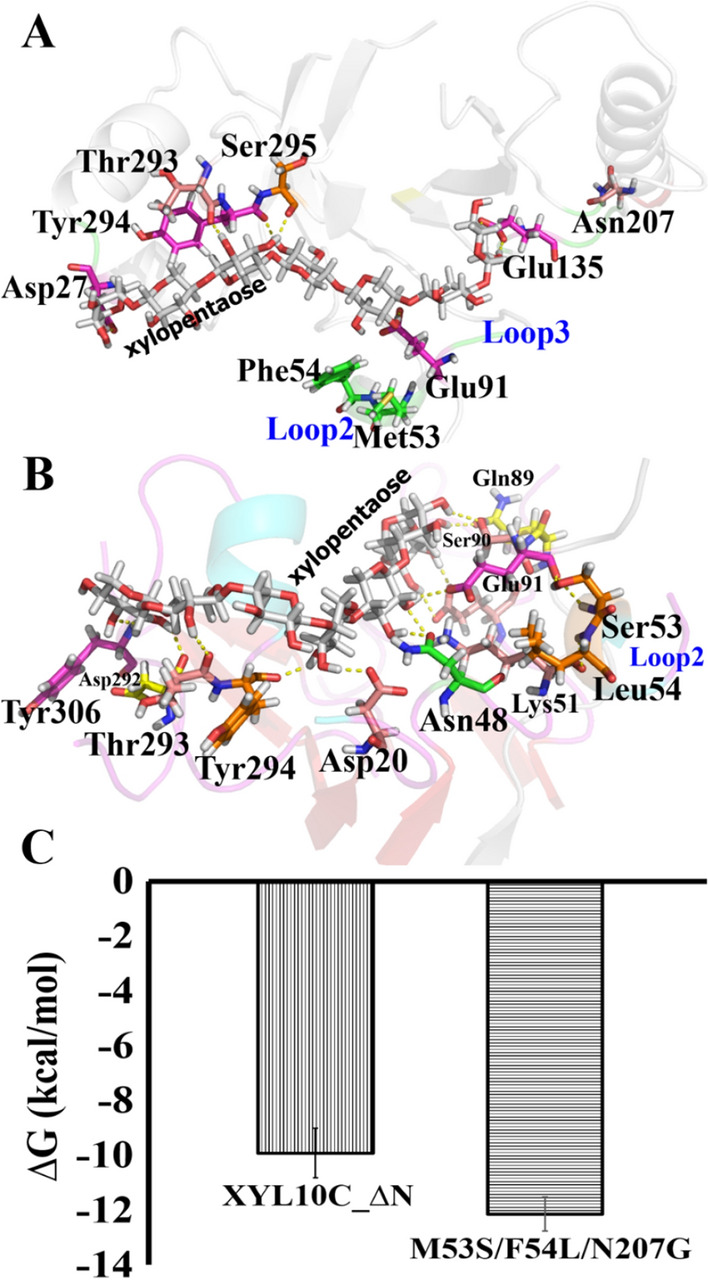
Structural analysis related to catalytic efficiency. Images **A** and **B** represent the confirmations of and interactions between xyloheptaose and the residues in the catalytic tunnel of XYL10C_∆N and its mutant M53S/F54L/N207G, respectively; **C** binding free energy values of XYL10C_∆N and the mutant M53S/F54L/N207G were calculated using the molecular mechanics Poisson–Boltzmann surface area

Early studies proposed that the catalytic cycle for enzymes with buried active sites comprises three major steps: substrate binding, enzyme catalysis, and product release [30, 31]. In the kinetic analysis, the decreased *K*_m_ and increased *k*_cat_ improved the catalytic activity of M53S/F54L/N207G. To explain the results of the conformation analysis, the structures of the XYL10C_∆N-xylopentaose complex and M53S/F54L/N207G-xylopentaose complex in the equilibrium state were investigated and are shown in Fig. 3. Lys51 and Glu91, which are two key amino acid residues in the catalytic pocket of GH10 xylanase, are closely associated with substrate binding. Their conformational stability facilitates substrate entry into the catalytic channel and completion of the catalytic process [17]. Ser53 and Leu54 on loop2 can form hydrogen bonds with Lys51 and Glu91, respectively (Fig. 3B). However, Met53 and Phe54 cannot form hydrogen bonds to interact with the adjacent Lys51 and Glu91 residues in the WT complex structure (Fig. 3A), which may be one of the reasons for the improved catalytic efficiency of mutant M53S/F54L/N207G. In addition, in the WT complex, the substrate can form hydrogen bonds with six residues in the catalytic pocket, whereas in the mutant complex, it can form hydrogen bonds with 11 residues, which causes a drastic increase in the enzyme–substrate binding force (Fig. 3B). The truncation [32] and replacement [33] of the inactive loop (surface loop) could alter the conformation of the active loop (catalytic loop). Thus, the catalytic channel is reconfigured to alter the catalytic efficiency and substrate selectivity of the enzymes.

Binding affinities were used to examine potential internal amino acid substitutions in the enzyme and its substrates to better understand the structural alterations that enhance the activity of M53S/F54L/N207G [34]. As shown in Fig. 3C, the binding affinities of mutant M53S/F54L/N207G were much lower than those of XYL10C_∆N (− 12.14 kcal/mol vs. − 9.92 kcal/mol), indicating the enhanced combination of the enzyme and the substrate, which improves the catalytic activity.

**Fig. 4:**
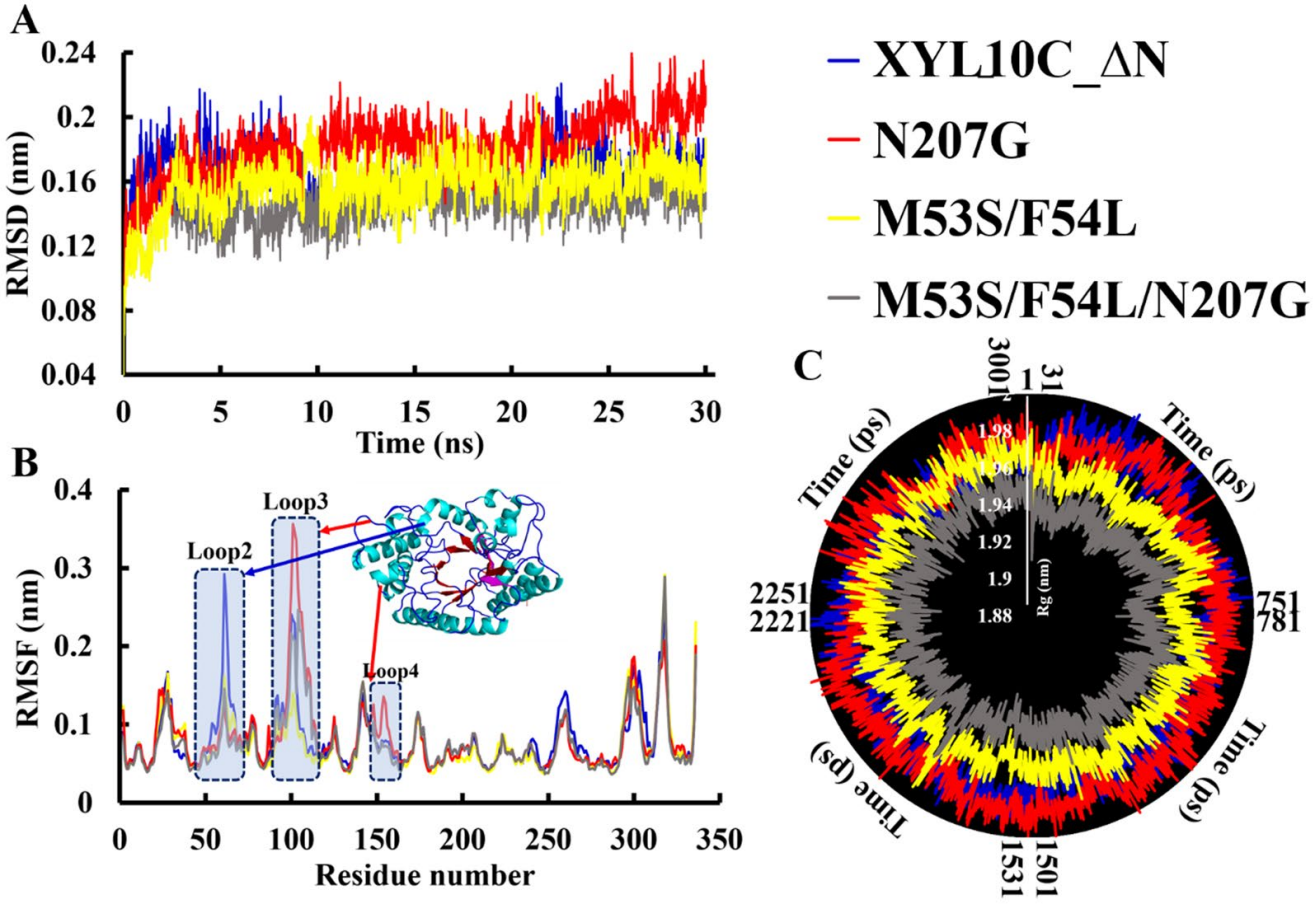
Molecular dynamics simulation analysis of thermal stability. **A** Root mean square deviation (RMSD) values for wild-type XYL10C_∆N and its mutants. **B** Root mean square fluctuation (RMSF) values during molecular dynamics (MD) simulation; **C** vibration deviation radius (Rg) of wild-type XYL10C_∆N and its mutants

MD simulations of the WT and its three mutants were performed for 30 ns at 313 K. As represented by the root mean square deviation (RMSD) of the protein backbone relative to the initial conformation, all systems reached a state of dynamic equilibrium after the first 10 ns of the simulation. Generally, the overall RMSD of the enzyme structure is reduced, making it rigid and, resultantly, increasing its thermostability [35]. Compared with the WT, the mutants M53S/F54L and M53S/F54L/N207G displayed enhanced conformational rigidity, with lower RMSD values at 313 K. However, the mutant N207G showed improved flexibility (Fig. 4A). Therefore, M53S/F54L and M53S/F54L/N207G were more thermostable than the WT. Moreover, as shown in Fig. 4B, the plasticity of all mutants was measured by root mean square fluctuation analysis and compared with that of the WT through independent trajectory calculations. Some regions, especially the loops between α-helices and β-sheets, showed significant differences in conformational fluctuations. For instance, the 53rd and 54th mutations enhanced the rigidity of loop2 (residues 48‒64), thereby enhancing the thermal stability of the protein. The N207G mutation mainly increased the flexibility of loop3 (residues 85–105) and loop4 (residues 133–156), thereby reducing the stability of the protein. “Rg” stands for the radius of rotation, which measures the degree of expansion and compression of the protein system. The smaller the radius, the smaller the expansion, and greater the protein stability [36]. The overall structures of M53S/F54L and M53S/F54L/N207G were tighter than those of the WT, and the mutant N207G was slightly swollen compared to the WT (Fig. 4C). The results of the simulations were consistent with the trend of the experimental data, indicating that the thermostability of the two mutants was significantly greater than that of the WT.

### Hydrolysis of various lignocellulosic substrates with cellulase and xylanase

**Fig. 5:**
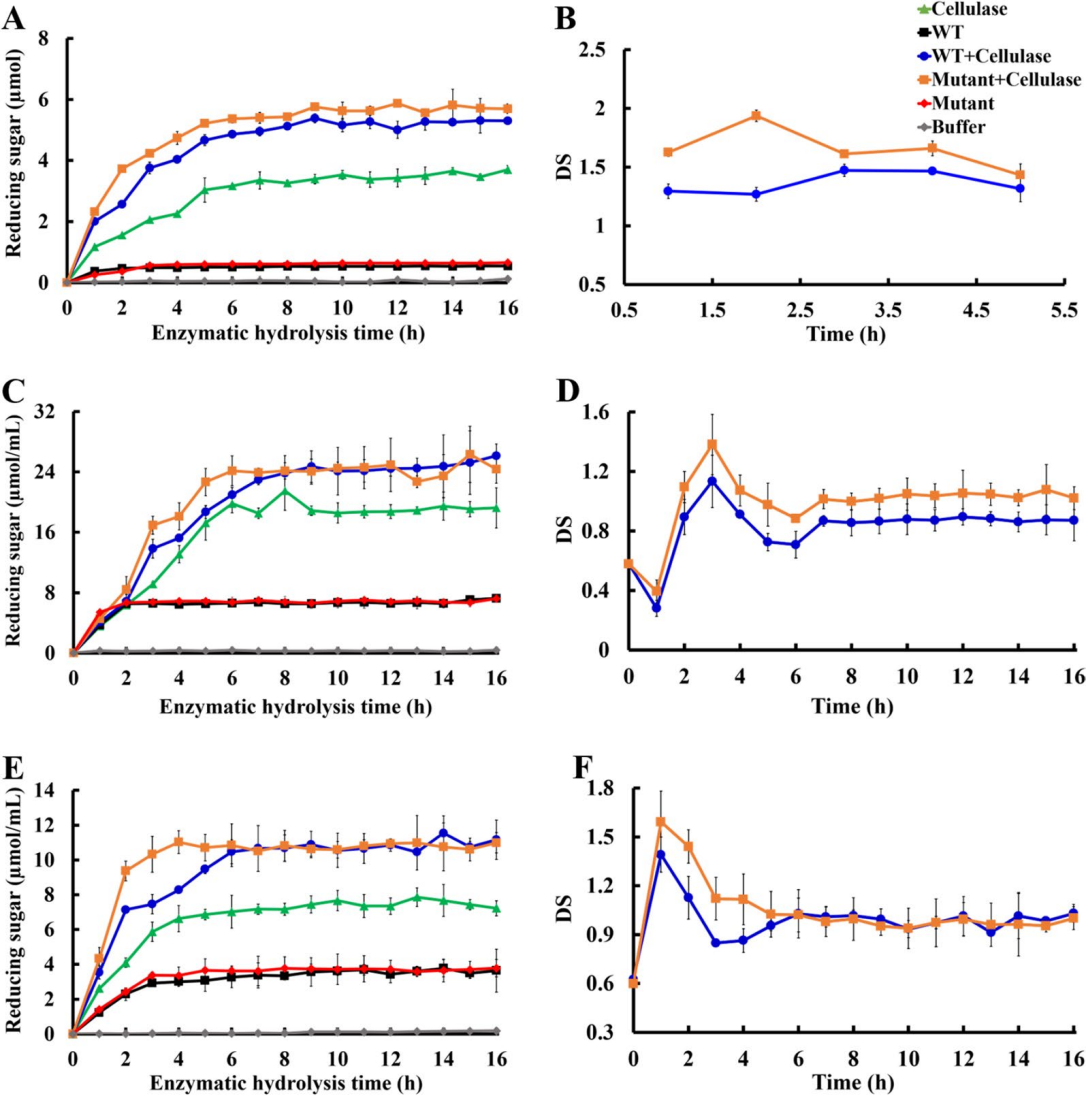
Time-course hydrolysis: corn stalk (**A**, **B**), wheat bran (**C**, **D**), and corn cob (**E**, **F**). **A**, **C**, and **E** Separate hydrolysis: 50 U cellulase (circle) or 50 U xylanase (triangle); simultaneous hydrolysis: 50 U cellulase and 50 U xylanase (inverted triangle); control: no enzyme added (square) to substrates for 16 h; **B**, **D**, and **F** degree of synergy curve of corn stalk (**B**), wheat bran (**D**), and corn cob (**F**). *WT* wild-type

In this study, xylanase (XYL10C_∆N and its mutant M53S/F54L/N207G) and cellulase were individually or simultaneously added to alkali-pretreated corn stalk, wheat bran, and corn cob to assess the generation of reducing sugars. Regardless of the enzyme components used to treat the three biomasses, the quantity of reducing sugars produced increased gradually till a stable plateau was reached (Fig. 5). When the mutant M53S/F54L/N207G hydrolyzed the three substrates individually, sugar production peaked with concentrations of 0.65, 6.7, and 3.4 µmol/mL at 4, 2, and 3 h, respectively, which was not significantly different from that achieved using WT XYL10C_∆N (Fig. 5A, C and E). The reducing sugar production peaked with concentrations of 3.2, 18.5, and 7.7 µmol/mL at 6, 7, and 10 h, respectively, when only cellulase was used to process the three substrates. When cellulase and the mutant M53S/F54L/N207G were used to synergistically hydrolyze the three substrates, the sugar production peaked with concentrations of 5.4, 24.3, and 11.0 µmol/mL at 6, 7, and 4 h, respectively. Compared with that in the WT co-treatment group, the time taken for the mutant xylanase co-treatment group to reach the maximum production was less by 1, 2, and 3 h. The rates of reducing sugar production in the first 2 h in the mutant co-treatment group were 45%, 24%, and 31% higher than those in the WT co-treatment group. In addition, the maximum sugar yield in the mutant co-treatment group was 59%, 35%, and 40% higher than that in the cellulase-only treatment group, and it was 8.0 times, 2.7 times, and 1.9 times higher than that in the xylanase treatment group.

The synergistic effects of xylanase on the cellulose-mediated hydrolysis of biomass were determined based on the degree of synergy (DS) (Fig. 5B, D and F). By definition, the quantity of reducing sugars produced upon the addition of xylanase with cellulase is associated with a larger DS. Large quantities of reducing sugars were produced by the addition of xylanase to cellulase. The DS values of the mutant cooperative treatment group were 1.9, 1.4, and 1.6, at 2, 3, and 1 h, respectively, which were the highest values achieved and 27%, 27%, and 14% higher than those of the WT cooperative treatment group, respectively. These results indicate that the xylanase mutants promoted cellulase-catalyzed hydrolysis of the three types of biomass, and the mutants could be arranged as M53S/F54L/N207G > XYL10C_∆N in decreasing order of strength. Previous studies have reported that xylanase can significantly promote cellulase degradation in bagasse. During alkaline pretreatment in bagasse hydrolysis, endoxylanase Xyn11A and endoglucanase Cel7B exhibited a strong synergistic effect (DS: 6.3), which significantly increased sugar production [37]. Therefore, the type of substrate and the pretreatment method used may influence the synergistic effect between xylanase and cellulase.

**Fig. 6:**
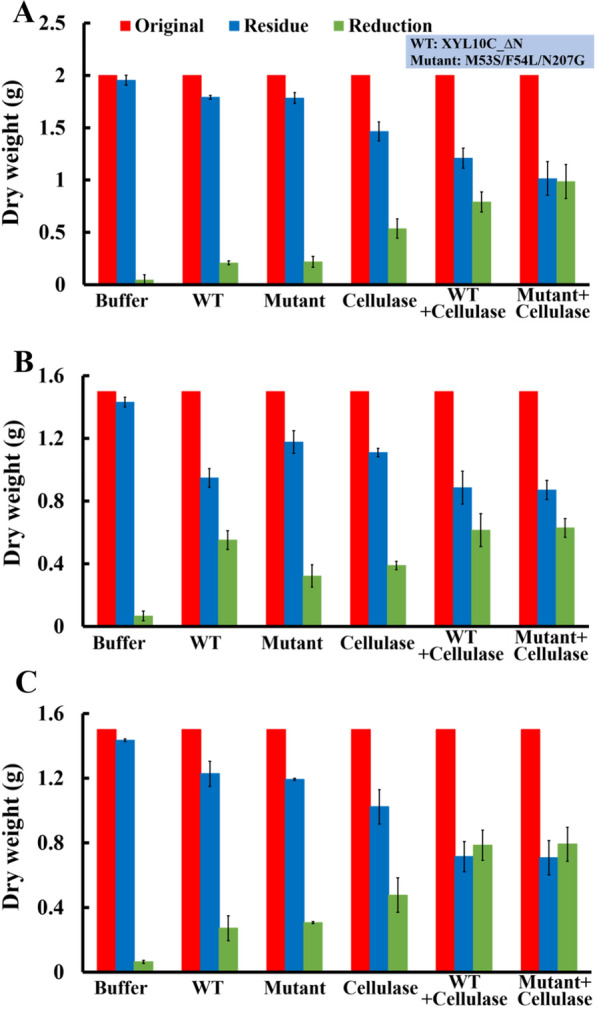
Changes in the dry weights of corn stalk, wheat bran, and corn cob. The changes that occurred during separate and simultaneous hydrolysis with cellulases and xylanase after 24 h were evaluated. **A** Corn stalk, **B** wheat bran, **C** corn cob. *WT* wild-type

The changes in the dry matter quality of the three substrates after hydrolysis were analyzed (Fig. 6). The dry matter reduction in the mutant cooperative treatment group was 0.64 g, 0.83 g, and 0.76 g after 24 h, accounting for 32%, 55%, and 51% of the total dry matter weight, respectively (Fig. 6). In comparison to that in the cellulase-only treatment group and xylanase-only treatment group, the dry matter reduction increased by 60%, 51%, and 58% and 3, 1.1, and 1.5 times, respectively, and there was no significant difference compared with the WT co-treatment group.

### Scanning electron microscopy (SEM)

**Fig. 7:**
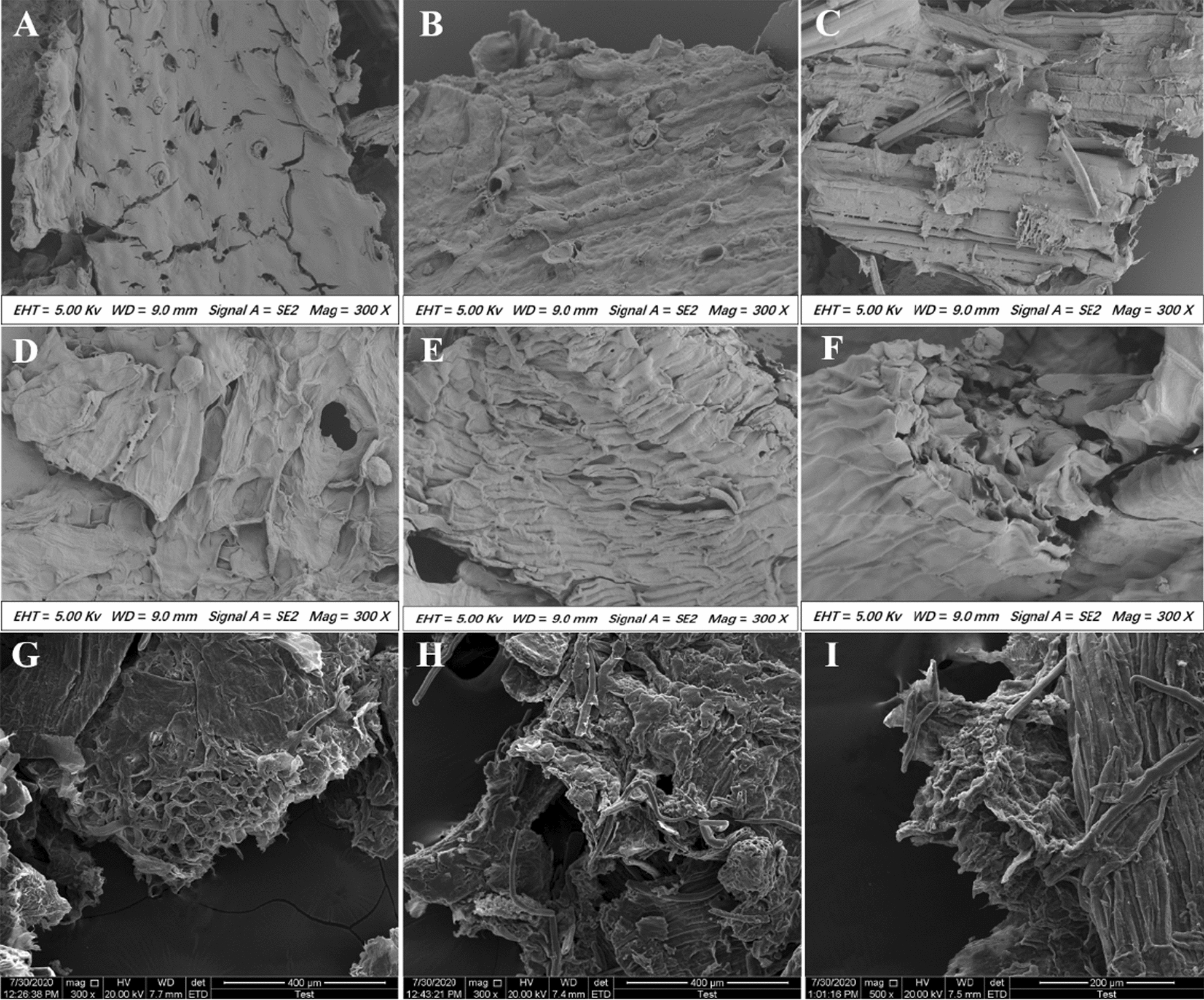
Electron microscopy scan showing the microstructures of the biomass treated with different enzymes. **A** Treatment of corn stalk with only MF53/54SL + N207G for 24 h; **B** treatment of corn stalk with only cellulose for 24 h; **C** simultaneous treatment of corn stalk with MF53/54SL + N207G and cellulase for 24 h. Magnification: 300 × for all images. **D**, **E**, and **F** and **G**, **H**, and **I** represent images of wheat bran and corn cob, respectively, subjected to the same treatments

SEM was used to observe the alterations in the surface structure of corn stalk, wheat bran, and corn cob after various enzyme treatments. Additional file 2: Figure S5 shows the surface structures of the three substrates after 24 h of buffer treatment. The results show that the surface structure of the plant-derived biomasses showed considerable alterations owing to the presence of cellulose, hemicellulose, lignin, and other major components in different proportions or owing to the different degrees of cross-linking. As shown in Fig. 7, xylanase treatment of the three substrates significantly changed the surface structure of the biomass. Numerous holes appeared on the surface of the corn stalks, which continued to enlarge (Fig. 7A). The cell wall structure on the surface of wheat bran was damaged, and the boundaries between the cells disappeared (Fig. 7D). The corn cob was first hydrolyzed internally, which was followed by the appearance of numerous holes (Fig. 7G). In comparison, cellulase-only treatment caused more severe damage to the surface of the biomass, such as deeper peeling between fibers, more severe fiber fragmentation, and formation of larger holes. However, cellulose is covered by a layer of hemicellulose, tightly interwoven with other components, and cannot be degraded extensively (Fig. 7B, E and H). When xylanase and cellulase synergistically hydrolyzed the three substrates, the degree of damage to the surface of the biomass increased, the interwoven state of each component was completely disrupted, and the pores disappeared completely. Moreover, the degree of adhesion of cellulose to hemicellulose was reduced (Fig. 7C, F and I). The cross-linking between cellulose and hemicellulose leads to the formation a complex network arrangement. Xylanase may destroy the original network structure of lignocellulose, thereby increasing the degree of swelling and porosity. Xylanase, as an auxiliary enzyme, damages the physical structure of lignocellulose to promote the penetration of cellulase into the microfibrous pores of cellulose and easier binding to cellulose, thereby accelerating substrate hydrolysis and improving reducing sugar yield [38]. The synergistic action of xylanase is a reliable strategy to overcome the obstacles in the saccharification reaction, and to eventually ensure the maximum reducing sugar yield from lignocellulosic substrates with the least enzyme titer.

## Conclusions

In this study, protein engineering was applied to obtain several mutants of xylanase, and the potential for industrial applications was evaluated. In this process, loop2 was found to be a key functional area affecting the low-temperature catalytic efficiency of GH10 xylanase. The mutant M53S/F54L/N207G emerged as the xylanase with the best low-temperature catalytic efficiency and residual thermostability, in accordance with the industrial demands. The selected mutant also showed excellent synergy with cellulase in the degradation of different types of lignocellulosic biomass. This study provides useful insights into the mechanisms and methods of xylanase modification for industrial utilization. However, future studies should investigate potential alterations in the loop2 conformation for improving the properties of xylanase and optimization of the enzymolysis strategy.

## Methods

### Materials

The gene encoding an objective-engineered xylanase ([WT] XYL10C_∆N, GenBank accession number FJ492963) with codon optimization was synthesized by Qingke Biotechnology (Nanjing, China). Competent *Escherichia coli* Trans1-T1 cells purchased from Tiangen (Nanjing, China) were used for plasmid DNA amplification. Vector pPIC9k and *Pichia pastoris* GS115 were purchased from Invitrogen (Carlsbad, CA, USA) and used for recombinant enzyme expression. FastPfu DNA polymerase, a Fast Mutagenesis System Kit, and restriction endonucleases (*E*coRI, *N*otI, and *B*glII) were procured from Vazyme (Nanjing, China). Standards and beechwood xylan were purchased from Sigma-Aldrich (St. Louis, MO, USA). Wheat bran, corn stalk, and corn cob were collected from farmlands in the Jingkou District of Zhenjiang City. All other reagents were of analytical grade and were commercially available.

### Selection of the mutation site and site-directed mutagenesis

Loop2 of XYL10C_∆N is located around the catalytic channel, and the component residue 52-FMFT-55 in loop2 forms a unique helical structure (Fig. 1). Multiple sequence alignments of fungal xylanases of GH10 were conducted using FASTA [39] and ClustalW algorithms [40]. Following conformational and sequence scrutiny of loop2, two key locations related to XYL10C_∆N functionality were identified and selected for mutagenesis. The mutants were first constructed using the Fast Mutagenesis System Kit (TransGen), using the recombinant plasmid pPIC9-XYL10C_∆N as a template for preliminary screening. All primers used in this study are listed in the Additional file 1: Table S1.

### Protein expression and purification

After verification of the DNA sequence, the recombinant plasmid was linearized using the restriction endonuclease *Bgl*II, and was further electroporated to transform *P. pastoris* GS115 host cells for recombinant gene expression. Screening of the positive transformants was conducted based on the activities of the enzymes in shake tubes, as described by Luo et al. [16], and the transformant with the highest xylanase activity at 40 °C was selected for fermentation. The transformants selected were precultured in yeast extract peptone dextrose (30 mL, 1% yeast extract, 2% tryptone, and 2% glucose) media overnight at 30 °C, with shaking at 220 rpm, and were then cultured for protein expression in a buffered glycerol-complex medium (300 mL, 1% glycerol, 2% tryptone, 1.34% yeast nitrogen base [YNB], and 4 mg/mL biotin) in a 1-L flask at 30 °C for 48 h, with shaking at 220 rpm. Protein expression was then induced by transferring the cells into a buffered methanol-complex medium (200 mL, 2% tryptone, 1.34% YNB, 4 mg/mL biotin, and 1% carbinol) in a 1-L flask at 30 °C and 220 rpm in a shaking incubator for 48 h. During the induction process, the medium was supplemented with 1% (v/v) carbinol every 12 h.

To separate and collect the crude protein supernatant, the fermentation broth was centrifuged at 12,000×*g* for 10 min. The ammonium sulfate precipitation method was used to concentrate the sample to 10 mL, following which the sample was dialyzed and desalted overnight in 20 mM McIlvaine buffer (pH 6.5). The desalted sample was then loaded onto a HiTrap SP HP 5-mL FPLC column (GE Healthcare) pre-equilibrated with buffer. Finally, the target protein was eluted using a linear gradient solution of NaCl (0–1.0 M) in the same buffer. SDS-PAGE was used to determine the purity of the target protein. For the estimation of protein concentration, the Bradford assay was performed, using bovine serum albumin as the standard.

### Determination of enzymatic properties

The 3,5-dinitrosalicylic acid method was used to detect the reducing sugars produced [45]. The enzyme titer required to release 1 μmol of reducing sugar per min under the test conditions was defined as one unit of enzyme activity (U). Glycine–HCl (100 mM, pH 1.0–2.5), citric acid–Na_2_HPO_4_ (200 mM, pH 2.5–8.0), and glycine–NaOH (100 mM, pH 8.0–11.0) were used to measure the pH adaptability and stability profiles. The optimal pH for enzymes was pH 1.0–9.0, as determined at 85 °C with reaction for 10 min. The pH stability of the enzymes was evaluated by pre-incubating the enzyme at 37 °C for 1 h without the substrate, followed by measurement of the residual activities at pH 4.0 and 85 °C.

The following four factors were used to compare the thermal properties of the enzymes: *T*_max_, *t*_1/2_ of the enzyme at a specific temperature, the temperature corresponding to 50% residual activity of the maximum activity after 30 min of treatment (*T*_50_), and the temperature corresponding to denaturation of 50% of the protein structure (*T*_m_). The *T*_max_ values for all enzymes were measured in the temperature range of 30–95 °C at the optimum pH for 10 min. The *t*_1/2_ values of the enzyme were measured residually under optimal conditions of temperature and pH and after incubation at 85 °C or 90 °C without the substrate at a concentration of approximately 50 µg/mL. In the absence of the substrate, the *T*_50_ values were determined after incubating the enzyme (approximately 50 µg/mL) for 0.5 h at the specified temperature range (between 75 and 95 °C). The *T*_m_ of the enzymes was assessed using a MicroCal™ VP-Capillary differential scanning calorimetry apparatus (GE Healthcare, Sweden) in 0.5 mL of 20 mM McIlvaine buffer (pH 6.5) at a concentration of approximately 0.2 mg/mL. After the protein and control were degassed, they were heat-treated in the range of 30–110 °C at a heating rate of 2 °C/min. All reactions were conducted and analyzed in triplicate.

The kinetic parameters (*K*_m_, *V*_max_, and *k*_cat_) of all purified enzymes were measured under low temperature (40 °C, optimal pH, for 5 min) and optimal conditions in 100 mM McIlvaine buffer containing beechwood xylan, sugarcane xylan, and corncob xylan (concentration: 0.5 mg/mL) as substrates. GraphPad Prism (version 5.01; La Jolla, CA, USA) and the Michaelis–Menten model were used to calculate the kinetic parameters. All tests and calculations were repeated three times.

### Molecular docking and MD simulation

To analyze the changes in the interaction between the enzyme and the substrate after mutation, the WT and its mutants were docked with xylopentaose using AutoDock Vina for theoretical rigid docking [41]. The docked enzyme–substrate complex underwent energy optimization and conformational screening. MD simulation was performed at 313 K for 30 ns using the Amber 14 package. The force field ff99SB was used to describe the system [42]. The RMSD values were assessed using minimum squares fitting of the protein backbone molecules. The first 20 ns simulation was used for the stabilization of the system, and the last 10 ns of the trajectory information was used for the dynamic simulation analysis. The best value was chosen using AutoDock Vina, considering the interaction energy, binding affinity (∆G, kcal/mol), and geometrical matching quality. Three-dimensional visualization and graphic preparation of protein molecules were completed using PyMOL version 1.7.2.1. All simulations were repeated three times.

### Enzymatic hydrolysis of lignocellulosic biomass

Corn stalk, wheat bran, and corn cob were soaked in 15% (w/w) aqueous ammonia at 60 °C for 24 h, as previously described [43]. Three lignocellulose biomass samples were soaked in 30 mL of phosphate buffer (pH 4.5, 0.01 mM) at a concentration of 5% (w/v), following which cellulase from *Aspergillus niger* [44] and/or xylanases were added. Subsequently, 30 mL of the reaction solution was placed in a 50-mL Erlenmeyer flask and incubated at 40 °C with shaking at 220 rpm for 24 h. The experiments were divided into five groups: cellulase (50 U)-added group, xylanase (50 U)-added group, both cellulase (50 U)- and xylanase (50U)-added group, including the WT and mutant groups, and a control group. The buffer volume loadings of the control group were the same as those of the experimental group. During enzymatic hydrolysis, the samples (0.8 mL) were collected in 1.5-mL Eppendorf tubes at regular intervals of 1 h. The supernatant was obtained from the mixture after centrifugation at 12,000×*g* for 5 min and subjected to further analysis. Triplicate experiments were performed in parallel.

The quantity of reducing sugar produced in the supernatant of the reaction system was calculated using the formula of the Lambert–Beer law:

1 $${\text{C}} = \frac{A}{\epsilon L},$$

where *A* is the absorbance and *ϵL* is the slope of the xylose standard curve. The DS value was calculated using the following equation:

2 $${\text{DS}} = \frac{{Y_{1 + 2} }}{{Y_{1} + Y_{2} }},$$

where $Y_{1 + 2}$ represents the content of reducing sugars produced in the reaction system when xylanase and cellulase were added simultaneously. $Y_{1}$ and $Y_{2}$ represent the quantity of reducing sugar when cellulase and xylanase are added during the hydrolysis process, respectively [43].

The data obtained from this experiment were analyzed using SPSS 17.0, one-way analysis of variance (ANOVA), and Origin Pro 9.

### SEM analysis

After the reaction was complete, an insoluble precipitate was collected. The soluble components were removed by washing three times with deionized water, followed by drying at 60 °C. Lastly, after sputtering gold plating on the dried sample, SEM was used to observe the changes in the surface structure of the sample at 300–400× magnification.

## Supplementary Information

**Additional file 1: Table S1. Primers used in this study.**

**Additional file 2: Figure S1.Analysis of multiple sequences of GH10 xylanases.** The selected mutation sites are marked with red diamonds. **Figure S2.** **Specific activity of wild-type XYL10C_∆N and its mutants against beechwood xylan at 40 ºC. Figure S3. Sodium dodecylsulfate-polyacrylamide gel electrophoresis analysis of purified XYL10C_∆N and its mutants. **Lanes: M, the standard protein molecular weight markers; **A, C, E, **and** G**: XYL10C_∆N, M53S/F54L, N207G, and M53S/F54L/N207G;** B, D, F, **and** H**: deglycosylated enzymes. **Figure S4. Graph for Lineweaver–Burk regression and equation for the enzymes at 40 °C. A.** XYL10C_∆N;** B** M53S/F54L;** C** N207G;** D** M53S/F54L/N207G**. Figure S5. **Surface structure of **A** corn stalk**, B** wheat bran, and **C** corn cob treated with buffer for 24 h.

## Data Availability

The dataset supporting the conclusions of this article is included within the article and its Additional files 1 and 2.

## Abbreviations

DS: Degree of synergy
GH: Glycoside hydrolase
MD: Molecular dynamics
RMSD: Root mean square deviation
SDS-PAGE: Sodium dodecyl sulfate-polyacrylamide gel electrophoresis
SEM: Scanning electron microscopy
*t*_1/2_: Half life
*T*_50_: Temperature corresponding to 50% residual activity of the maximum activity after 30 min of treatment
*T*_m_: Melting temperature
*T*_max_: Temperature corresponding to the maximum enzyme activity
YNB: Yeast nitrogen base W/O amino acids
