# Supplementary material for: Improvement of XYL10C_∆N catalytic performance through loop engineering for lignocellulosic biomass utilization in feed and fuel industries
Source: Biotechnol Biofuels. 2021 Oct 1;14:195. doi: 10.1186/s13068-021-02044-3 (PMC8487158; doi:10.1186/s13068-021-02044-3)
Supplement: Supplementary file 1 — Additional file 1: Table S1. Primers used in this study. [file 13068_2021_2044_MOESM1_ESM.docx]

**Additional file**

**Improvement of XYL10C_∆N catalytic performance through loop engineering for lignocellulosic biomass utilization in feed and fuel industries**

Shuai You^1,2^, Ziqian Zha^1^, Jing Li^3^, Wenxin Zhang^1^, Zhiyuan Bai^1^, Yanghao Hu^1^, Xue Wang^1^, Yiwen Chen^1^, Zhongli Chen^4^, Jun Wang^1,2^*, Huiying Luo^5^*

^1^ School of Biotechnology, Jiangsu University of Science and Technology, Zhenjiang 212018, P R China;

^2^ Sericultural Research Institute, Chinese Academy of Agricultural Sciences, Zhenjiang 212018, P R China;

^3^ Department of Nephrology, Affiliated Hospital of Jiangsu University, Zhenjiang 212001, P R China;

^4^ Xinyuan cocoon silk group co., ltd., Nantong 226600, P R China;

^5^ Institute of Animal Sciences, Chinese Academy of Agricultural Sciences, Beijing 100081, China.

***Correspondence:** Jun Wang and Huiying Luo

*Phone*: +86-511-85635867, *Fax*: +86-511-85620901.

*E-mail*: [wangjun@just.edu.cn](mailto:wangjun@just.edu.cn) (Prof. Dr. Jun Wang); luohuiying@caas cn (Prof. Dr. Huiying Luo)

^1^ School of Biotechnology, Jiangsu University of Science and Technology, Zhenjiang 212018, People’s Republic of China

^5^ Institute of Animal Sciences, Chinese Academy of Agricultural Sciences, Beijing 100081, China.

**Additional file 1:**

**Table S1. Primers used in this study.^a^**

| **Primer name** | **Primer sequence (5′→3′) ^a^** | **Size (bp)** |
| --- | --- | --- |
| XYL10C_∆N-PF | gtagaattctggggtcttaataatgcag | 28 |
| XYL10C_∆N-PR | ttcgcggccgctcatggactttccgcct | 28 |
| MF53/54SL-F | atgaaattcagtttgacggagccagagc | 28 |
| MF53/54SL-R | caaactgaatttcataatattcgcgggt | 28 |
| N207G-F | cttaaagcaaggggcatccaaatagatg | 28 |
| N207G-R | gccccttgctttaagctccttgacaata | 28 |
| MF53/54AA-F | atgaaattcgccgccacggagccag | 25 |
| MF53/54AA-R | ggcggcgaatttcataatattcgcg | 25 |
| MF53/54VS-F | atgaaattcgtcagcacggagccag | 25 |
| MF53/54VS-R | gctgacgaatttcataatattcgcg | 25 |
| MF53/54ES-F | atgaaattcgaaagcacggagccagag | 26 |
| MF53/54ES-R | gctttcgaatttcataatattcgcg | 25 |
| MF53/54IS-F | atgaaattcatcagcacggagccagag | 27 |
| MF53/54IS-R | gctgatgaatttcataatattcgcgg | 26 |
| MF53/54ER-F | tatgaaattcgaacgcacggagccaga | 27 |
| MF53/54ER-R | gcgttcgaatttcataatattcgcggg | 26 |
| MF53/54SY-F | tatgaaattcagctacacggagccagag | 28 |
| MF53/54SY-R | gtagctgaatttcataatattcgcggg | 27 |
| MF53/54VV-F | cgcgaatattatgaaattcgtcgtcacggagcca | 34 |
| MF53/54VV-R | aattaaaaacgttttgctctggctccgtgacgac | 34 |
| MF53/54GV-F | atgaaattcggagtcacggagccagagcaaa | 31 |
| MF53/54GV-R | gactccgaatttcataatattcgcggg | 27 |
| MF53/54MY-F | tatgaaattcatgtacacggagccagagcaa | 31 |
| MF53/54MY-R | gtacatgaatttcataatattcgcggg | 27 |
| MF53/54FA-F | ttatgaaattcttcgccacggagccagagc | 30 |
| MF53/54FA-R | ggcgaagaatttcataatattcgcgggtgt | 30 |
| MF53/54PA-F | tatgaaattcccagccacggagccaga | 27 |
| MF53/54PA-R | ggctgggaatttcataatattcgc | 24 |
| MF53/54AE-F | tatgaaattcgccgaaacggagccagagcaa | 31 |
| MF53/54PAER | ttcggcgaatttcataatattcgcgggtg | 29 |

^a^ The restriction sites are underlined.
