# Supplementary material for: Improvement of XYL10C_∆N catalytic performance through loop engineering for lignocellulosic biomass utilization in feed and fuel industries
Source: Biotechnol Biofuels. 2021 Oct 1;14:195. doi: 10.1186/s13068-021-02044-3 (PMC8487158; doi:10.1186/s13068-021-02044-3)
Supplement: Supplementary file 2 — Additional file 2: Figure S1.Analysis of multiple sequences of GH10 xylanases. The selected mutation sites are marked with red diamonds. Figure S2. Specific activity of wild-type XYL10C_∆N and its mutants against beechwood xylan at 40 ºC. Figure S3. Sodium dodecylsulfate-polyacrylamide gel electrophoresis analysis of purified XYL10C_∆N and its mutants. Lanes: M, the standard protein molecular weight markers; A, C, E, and G: XYL10C_∆N, M53S/F54L, N207G, and M53S/F54L/N207G; B, D, F, and H: deglycosylated enzymes. Figure S4. Graph for Lineweaver–Burk regression and equation for the enzymes at 40 °C. A. XYL10C_∆N; B M53S/F54L; C N207G; D M53S/F54L/N207G. Figure S5. Surface structure of A corn stalk, B wheat bran, and C corn cob treated with buffer for 24 h. [file 13068_2021_2044_MOESM2_ESM.docx]

**Additional file 2：**


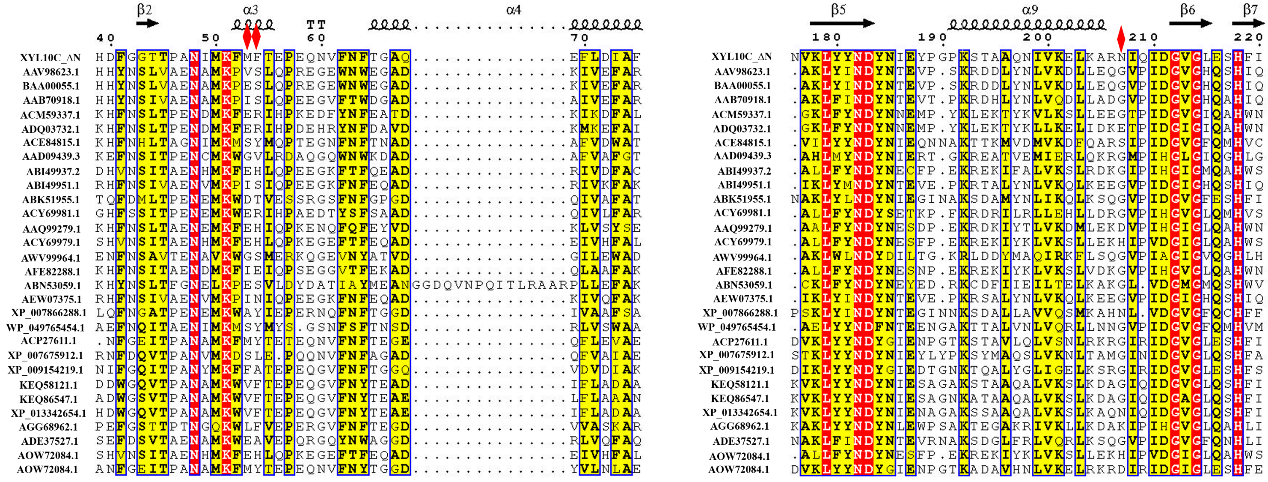


**Figure S1.** **Analysis of multiple sequences of GH10 xylanases.** The selected mutation sites are marked with red diamonds.


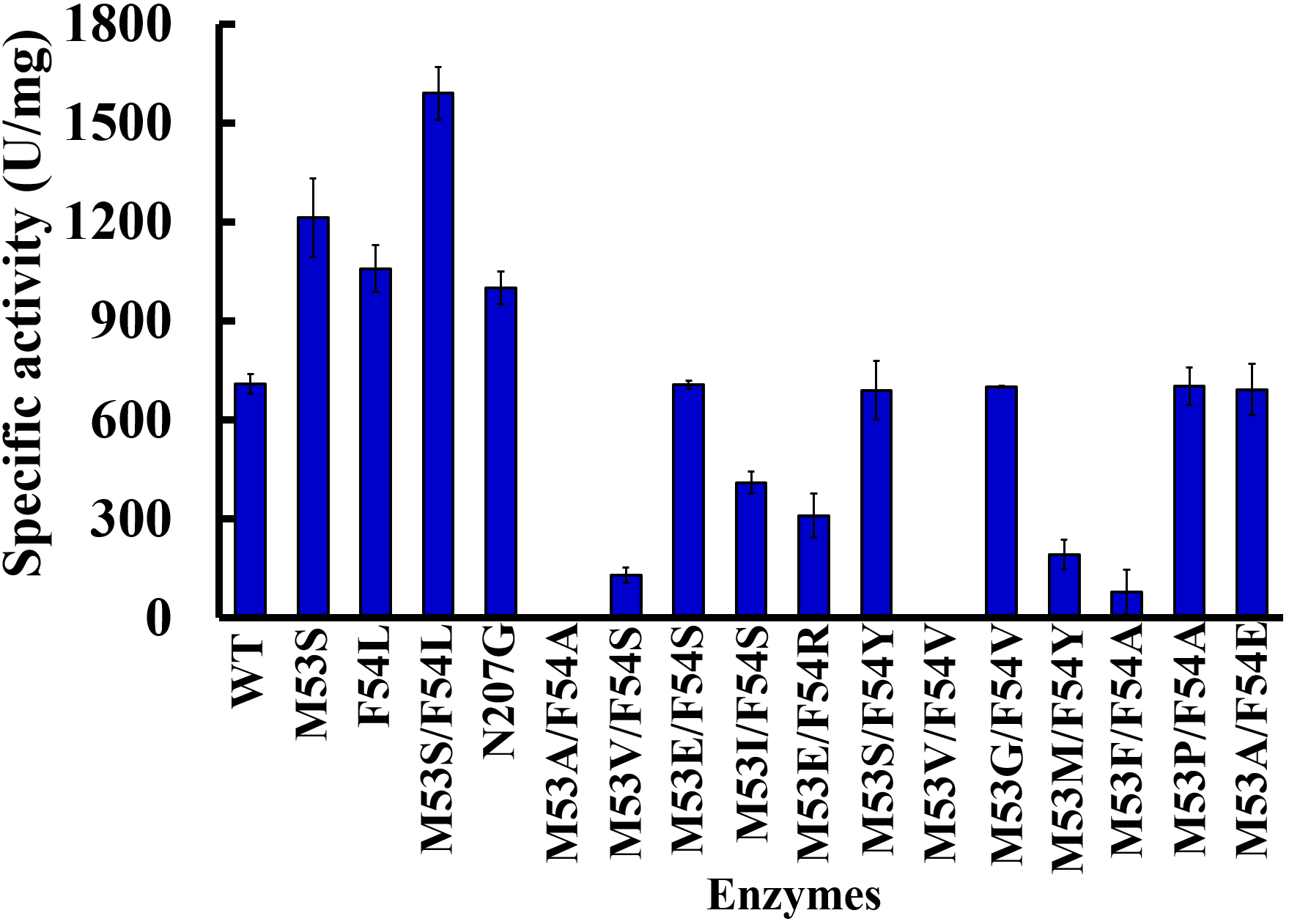


**Figure S2.** **Specific activity of wild-type XYL10C_∆N and its mutants against beechwood xylan at 40 ºC.**


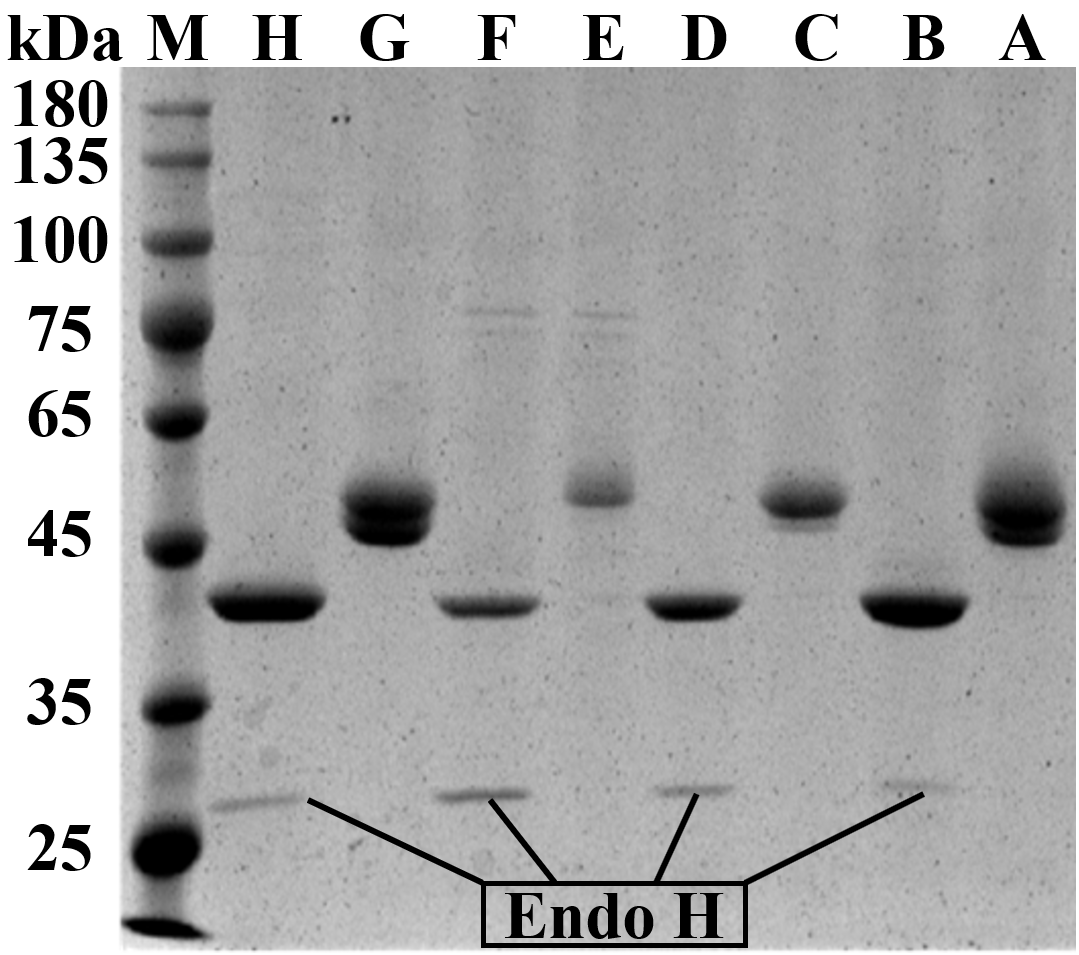


**Figure S3. Sodium dodecylsulfate-polyacrylamide gel electrophoresis analysis of purified XYL10C_∆N and its mutants.** Lanes: M, the standard protein molecular weight markers; **A, C, E,** and **G**: XYL10C_∆N, M53S/F54L, N207G, and M53S/F54L/N207G; **B, D, F,** and **H**: deglycosylated enzymes.


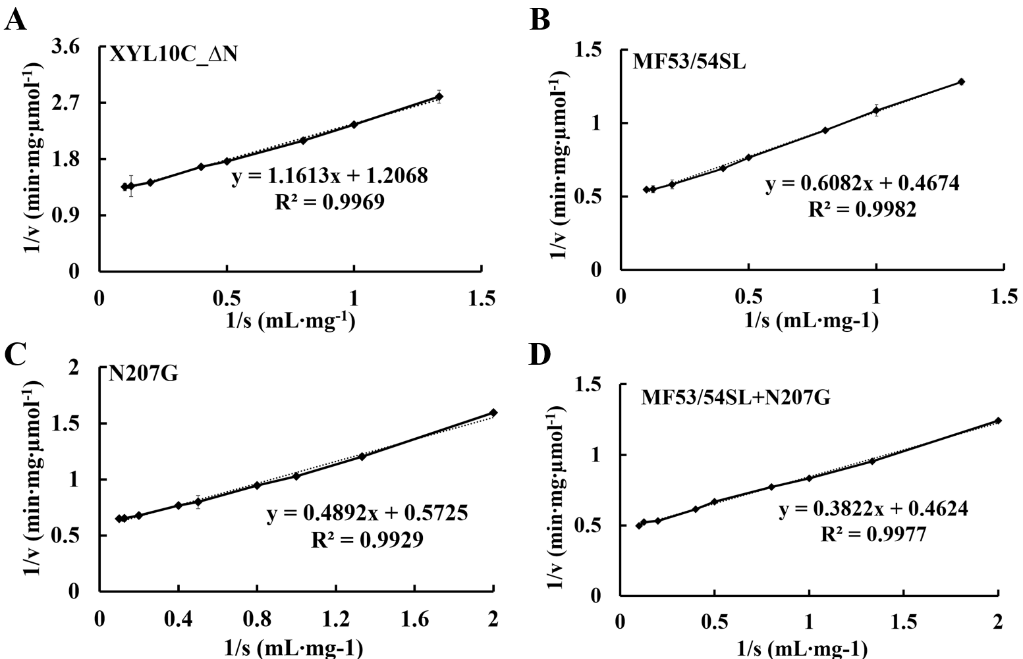


**Figure S4. Graph for Lineweaver–Burk regression and equation for the enzymes at 40 °C. A.** XYL10C_∆N; **B.** M53S/F54L; **C.** N207G; **D.** M53S/F54L/N207G**.**


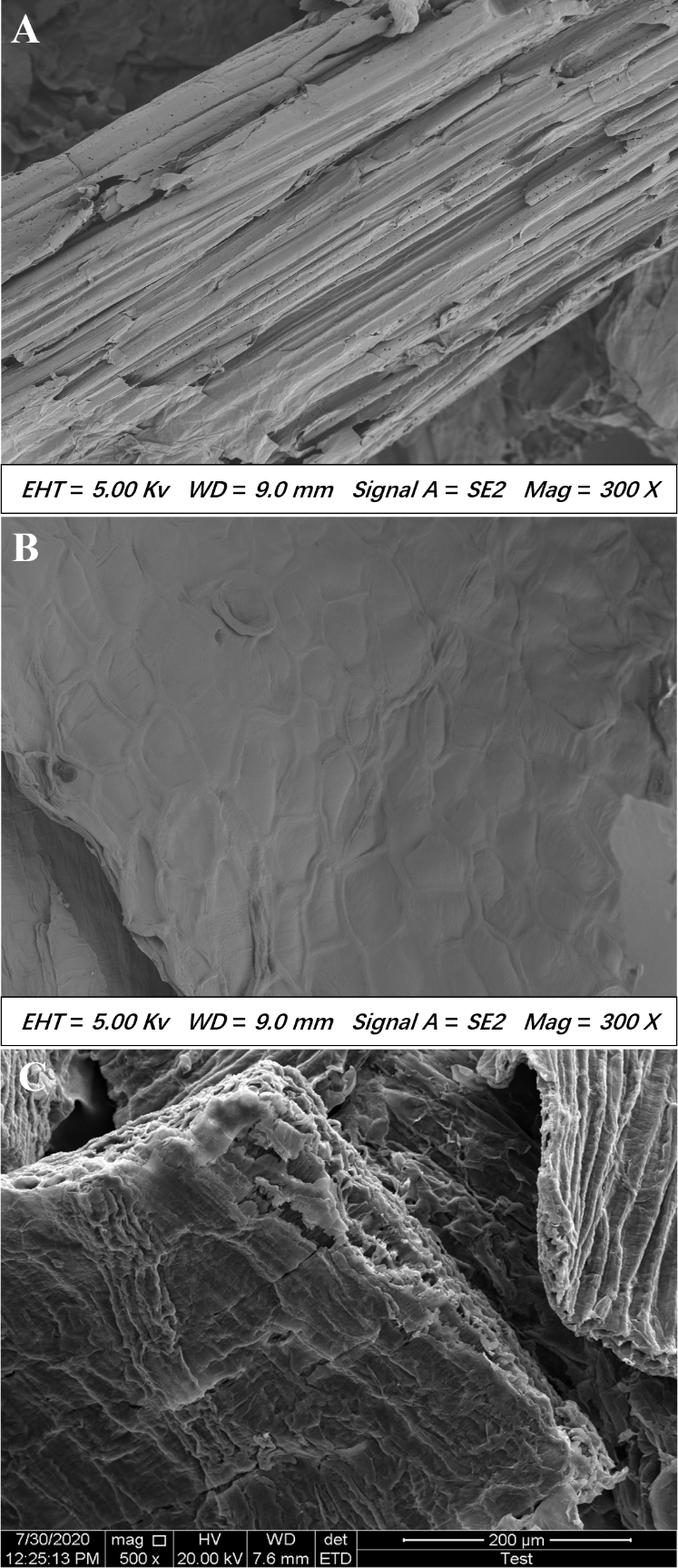


**Figure S5.** Surface structure of **A.** corn stalk**, B.** wheat bran, and **C.** corn cob treated with buffer for 24 h.
